# Ticagrelor-Associated Central Sleep Apnea: Mechanisms, Clinical Evidence, and Implications for Sleep Medicine

**DOI:** 10.7759/cureus.119361

**Published:** 2026-11-09

**Authors:** Binh Pham

**Affiliations:** 1 Psychiatry, Western University of Health Sciences, Pomona, USA; 2 Sleep Medicine, Amen Clinics, Costa Mesa, USA; 3 Sleep Medicine, Southwest Healthcare Medical Education Consortium, Temecula, USA

**Keywords:** adenosine, central sleep apnea, cheyne-stokes respiration, loop gain, p2y12 inhibitors, periodic breathing, respiratory control, sleep-disordered breathing, ticagrelor, ventilatory chemosensitivity

## Abstract

Ticagrelor is a reversible P2Y12 receptor antagonist used for antiplatelet therapy and is well recognized to cause dyspnea. Emerging concern has focused on a possible association between ticagrelor and central sleep apnea, Cheyne-Stokes respiration, and other manifestations of ventilatory instability. This narrative review examines the clinical evidence for ticagrelor-associated central sleep apnea and the respiratory-control mechanisms proposed to underlie this association. A structured literature search was performed using PubMed, with Google Scholar and citation tracking used for supplementary discovery. Human clinical, physiologic, pharmacovigilance, and mechanistic evidence was synthesized qualitatively according to study design and evidentiary strength. Overall, the literature supports a possible association between ticagrelor exposure and central respiratory instability, although the incidence, clinical significance, and susceptibility factors remain uncertain. Enhanced ventilatory chemosensitivity and ventilatory overshoot provide a plausible physiologic framework, whereas equilibrative nucleoside transporter 1 inhibition and altered adenosine signaling remain proposed rather than established mechanisms in humans. Ticagrelor-associated central sleep apnea should therefore be regarded as an emerging medication-associated respiratory phenotype requiring cautious clinical interpretation. Prospective studies using standardized respiratory phenotyping, appropriate comparator groups, serial assessment, and clinically meaningful outcomes are needed to clarify susceptibility, reversibility, mechanisms, and clinical consequences.

## Introduction and background

Central sleep apnea (CSA) is characterized by recurrent reductions or cessations of ventilation during sleep associated with diminished or absent respiratory effort [1]. The transition from wakefulness to sleep reduces the wakefulness-related contribution to ventilatory drive and increases dependence on metabolic feedback, particularly the arterial partial pressure of carbon dioxide (PaCO₂). In susceptible individuals, hyperventilation may reduce PaCO₂ below the apneic threshold, the level of carbon dioxide below which respiratory motor output is transiently suppressed, until carbon dioxide rises sufficiently to restore ventilation. The stability of this feedback system is commonly described using the concept of loop gain, which reflects the responsiveness of the respiratory control system to a disturbance, with increased loop gain favoring oscillation between hyperventilation and central apnea or hypopnea. CSA is mechanistically heterogeneous; hypocapnic ventilatory instability, in which excessive reductions in PaCO₂ destabilize breathing, represents one pathway, whereas other forms may result from impairment of respiratory rhythm generation within the brainstem [1].

Medication-induced CSA represents a clinically important but heterogeneous form of central sleep-disordered breathing. Opioids and several non-opioid medications have been associated with CSA through differing effects on respiratory rhythm generation and ventilatory control [2]. Ticagrelor is unusual in this context because its proposed effect does not primarily involve conventional respiratory suppression; rather, increased chemosensitivity and ventilatory responsiveness have been implicated in the development of periodic breathing and central respiratory events [2]. Comparative human investigation has demonstrated greater hypercapnic chemosensitivity, or ventilatory responsiveness to increased carbon dioxide, among patients receiving ticagrelor than among those receiving prasugrel [3]. Prospective physiologic testing has also demonstrated increases in both hypoxic and hypercapnic ventilatory responses following ticagrelor initiation that were not observed with clopidogrel [4]. These findings provide a physiologic basis for considering ticagrelor-associated CSA as a manifestation of ventilatory instability rather than conventional medication-induced respiratory depression.

Ticagrelor is an oral, direct-acting, reversible P2Y12 receptor antagonist used as antiplatelet therapy in acute coronary syndromes [5]. Dyspnea is a well-recognized adverse effect and occurred more frequently with ticagrelor than with clopidogrel in the PLATO trial [6]. The mechanism underlying this respiratory symptom remains incompletely defined, prompting investigation of altered chemoreflex function and respiratory control. In 2016, Giannoni et al. described Cheyne-Stokes respiration and increased chemoreflex sensitivity in association with ticagrelor-related dyspnea, providing an early clinical link between ticagrelor exposure and central respiratory instability [7]. In 2018, Revol et al. subsequently identified a disproportionate pharmacovigilance reporting signal for sleep apnea among ticagrelor users [8]. Together, these observations expanded the clinical signal from ticagrelor-associated dyspnea alone to a possible medication-associated disturbance of respiratory control during sleep.

The clinical evidence has since expanded beyond isolated observations. Reductions in central respiratory events following ticagrelor withdrawal and recurrence after reintroduction have provided dechallenge-rechallenge evidence that strengthens causal inference [9]. Comparative physiologic investigation subsequently demonstrated higher apnea-hypopnea and central apnea indices, together with greater hypercapnic chemosensitivity, among ticagrelor-treated patients compared with patients receiving prasugrel [3]. A prospective study after acute coronary syndrome identified predominantly CSA more frequently among patients receiving ticagrelor [10], while a later multicenter retrospective polysomnographic study found a greater central apnea burden among ticagrelor-treated patients than among those receiving clopidogrel [11]. However, the available evidence is not uniformly concordant. An analysis of large randomized, blinded ticagrelor trials did not identify an excess of reported sleep apnea with ticagrelor treatment [12]. The literature therefore spans dechallenge-rechallenge observations, pharmacovigilance data, physiologic studies, prospective and retrospective observational cohorts, and differing methods of respiratory-event ascertainment, requiring careful attention to study design when interpreting the strength of the association.

This review examines the clinical evidence regarding ticagrelor-associated CSA and evaluates the respiratory-control mechanisms proposed to underlie this association. Particular attention is given to ventilatory chemosensitivity, hypocapnic ventilatory instability, loop gain, adenosine-related signaling, and the potential relationship between ticagrelor-associated dyspnea and central respiratory events. Human clinical and physiologic findings are considered according to study design and evidentiary strength, with observed associations distinguished from proposed pharmacologic mechanisms and mechanistic plausibility distinguished from mechanisms demonstrated in humans. The review further considers the implications of this association for the recognition, evaluation, and management of medication-associated CSA in sleep medicine.

## Review

Literature search strategy

A narrative review of the literature was conducted to identify human clinical and physiologic evidence regarding ticagrelor-associated CSA and to provide the respiratory-physiology and pharmacologic context necessary for interpretation of these findings. PubMed was searched through September 2026 without restriction on publication date, and searches were limited to English-language publications. Google Scholar was used as a supplementary discovery and citation-tracking tool rather than as an exhaustive bibliographic database. Reference lists of relevant primary studies and review articles were also manually examined to identify additional publications.

For PubMed, the principal search for direct human evidence used the following search string: ("ticagrelor" OR "AZD6140") AND ("central sleep apnea" OR "central sleep apnoea" OR "central apnea" OR "central apnoea" OR "sleep apnea" OR "sleep apnoea" OR "Cheyne-Stokes respiration" OR "periodic breathing" OR "sleep-disordered breathing"). A supporting physiologic search used ("ticagrelor" OR "AZD6140") AND ("chemosensitivity" OR "chemoreflex" OR "hypercapnic ventilatory response" OR "hypoxic ventilatory response" OR "ventilatory response" OR "respiratory control" OR "ventilatory instability" OR "loop gain"). A mechanistic search used ("ticagrelor" OR "AZD6140") AND ("adenosine" OR "ENT1" OR "equilibrative nucleoside transporter 1" OR "P2Y12" OR "carotid body"). No field restrictions were applied. Additional targeted searches addressing medication-induced CSA, ticagrelor-associated dyspnea, and central respiratory-control physiology were used to provide broader clinical and mechanistic context.

Google Scholar was searched using targeted combinations of the same concepts, including "ticagrelor" "central sleep apnea", "ticagrelor" "central sleep apnoea", "ticagrelor" "Cheyne-Stokes respiration", "ticagrelor" chemosensitivity, "ticagrelor" chemoreflex, "ticagrelor" "periodic breathing", and combinations of ticagrelor with adenosine and ENT1. Because Google Scholar dynamically ranks search results, it was not treated as an exhaustive database search. The exact number of Google Scholar results screened was not prospectively recorded, and no predefined stopping rule was applied. Its role was therefore limited to identifying potentially relevant publications and supporting backward and forward citation tracking.

For the direct human evidence, publications were considered if they involved ticagrelor exposure and reported central respiratory events, Cheyne-Stokes respiration or periodic breathing, objective sleep or respiratory monitoring, respiratory changes following ticagrelor withdrawal or re-exposure, ventilatory chemosensitivity, or pharmacovigilance findings related to sleep apnea. Studies addressing ticagrelor-associated dyspnea without objective characterization of central respiratory abnormalities and studies examining related respiratory-control phenomena outside sleep were considered separately as contextual evidence when relevant.

Pharmacologic, physiologic, and broader CSA literature was considered separately when it addressed predefined areas relevant to interpretation of the human findings, including ventilatory chemosensitivity, hypocapnic respiratory instability, ENT1-mediated adenosine transport, and medication-induced CSA. Primary experimental and clinical studies were preferentially used for specific pharmacologic or mechanistic claims, whereas authoritative reviews and clinical guidelines were used primarily for broader synthesis and context. An evidentiary hierarchy was applied in which objective human sleep and respiratory findings were given the greatest weight for characterizing ticagrelor-associated central respiratory abnormalities. Dechallenge-rechallenge observations were given particular weight for individual-level causal inference, human physiologic evidence was used to interpret respiratory-control effects, and pharmacologic evidence was used to provide biological plausibility rather than establish mechanisms in humans.

Titles and abstracts were reviewed for relevance, followed by full-text assessment of potentially eligible human publications when necessary. Formal counts of records identified, screened, and excluded were not prospectively recorded because this review was designed as a narrative rather than systematic review, and Google Scholar was used only for supplementary discovery and citation tracking. The principal direct human evidence ultimately synthesized comprised 11 clinical, pharmacovigilance, and physiologic studies. Extracted information included study population, sample size, study design, cardiovascular context, ticagrelor exposure and comparator when applicable, respiratory assessment method, central respiratory outcomes, ventilatory chemosensitivity, withdrawal or re-exposure findings, and major study limitations. Methodological characteristics were evaluated qualitatively with attention to design-specific sources of bias, including small sample size, cardiovascular confounding, treatment-selection bias, differences in respiratory ascertainment, spontaneous-reporting bias, and whether measured outcomes represented CSA itself or physiologic correlates of respiratory instability. No formal risk-of-bias instrument was applied.

Because the available human evidence differed substantially in study population, design, respiratory-assessment method, comparator conditions, and outcome definitions, quantitative pooling was not considered appropriate. The findings were therefore synthesized qualitatively. Across studies, findings were compared according to study design, patient population, cardiovascular context, comparator treatment, respiratory-assessment method, outcome definition, evidentiary strength, and design-specific limitations, with greater weight given to objective human respiratory findings than to indirect or nonspecific outcomes. This article was designed as a narrative review rather than a systematic review or meta-analysis. Restriction to English-language publications, reliance primarily on PubMed, and supplementary use of dynamically ranked Google Scholar results may have introduced language, database-selection, and discretionary-selection bias and may have resulted in omission of relevant evidence. These limitations are addressed further in the Limitations and Future Directions section.

Respiratory control and ticagrelor pharmacology

Ventilation during sleep is regulated largely through metabolic feedback from changes in arterial carbon dioxide and oxygen tension. The transition from wakefulness to sleep reduces the wakefulness-related contribution to respiratory drive and increases dependence on chemoreceptor-mediated control of ventilation [1]. Carbon dioxide plays a particularly important role because a reduction in the PaCO₂ below an individual’s apneic threshold can transiently suppress respiratory motor output and produce central apnea [1]. The difference between the prevailing PaCO₂ and the apneic threshold, often referred to as the CO₂ reserve, therefore influences susceptibility to central respiratory events during sleep [1]. Individuals whose PaCO₂ lies relatively close to the apneic threshold may be particularly vulnerable to perturbations that increase ventilation and drive PaCO₂ downward. This relationship provides the physiologic basis for understanding how excessive ventilatory responsiveness, rather than respiratory suppression alone, can contribute to CSA.

The propensity for PaCO₂ to cross the apneic threshold depends not only on the CO₂ reserve but also on the responsiveness and stability of the ventilatory control system. Chemosensitivity describes the magnitude of the ventilatory response to changes in carbon dioxide or oxygen, whereas loop gain reflects the overall responsiveness of the respiratory feedback system to a disturbance [1]. When ventilatory responsiveness is excessive, a relatively small perturbation may provoke disproportionate hyperventilation, producing an overshoot that lowers PaCO₂ below the apneic threshold [1]. The resulting central apnea allows carbon dioxide to accumulate until ventilation resumes, after which another excessive ventilatory response may again produce hypocapnia. Repetition of this cycle can generate oscillatory breathing patterns ranging from recurrent central apneas to periodic breathing and Cheyne-Stokes respiration [1]. Increased chemosensitivity may therefore promote central respiratory instability by amplifying ventilatory responses to relatively small physiologic disturbances.

Ticagrelor is a direct-acting, reversible antagonist of the platelet P2Y12 receptor and differs pharmacologically from thienopyridine P2Y12 inhibitors such as clopidogrel and prasugrel [5]. In addition to P2Y12 inhibition, ticagrelor inhibits equilibrative nucleoside transporter 1 (ENT1), which participates in cellular uptake of adenosine [13]. Experimental pharmacologic studies have demonstrated inhibition of adenosine uptake through ENT1 by ticagrelor at clinically relevant concentrations, an effect not demonstrated with the active metabolites of clopidogrel or prasugrel [13]. By limiting cellular uptake, ENT1 inhibition may increase or prolong extracellular adenosine signaling. Because adenosine can influence sensory and chemoreflex pathways involved in respiratory regulation, this pharmacologic property has been proposed as one potential link between ticagrelor and altered ventilatory control. However, ENT1 inhibition alone does not establish that adenosine signaling mediates ticagrelor-associated CSA in humans.

Human physiologic studies provide more direct evidence that ticagrelor can alter ventilatory responsiveness. Giannoni et al. reported greater hypercapnic chemosensitivity in patients receiving ticagrelor than in those receiving prasugrel, together with a greater burden of central respiratory events [3]. Tubek et al. subsequently assessed chemoreflex responses before and after initiation of P2Y12 inhibition and found increases in both hypoxic and hypercapnic ventilatory responses following ticagrelor treatment, whereas comparable changes were not observed with clopidogrel [4]. These findings are consistent with increased chemoreflex responsiveness during ticagrelor exposure and provide a physiologic bridge between the drug’s pharmacology and susceptibility to ventilatory overshoot. Importantly, increased chemosensitivity is not equivalent to CSA, and these physiologic observations do not establish which pharmacologic pathway is responsible for the altered respiratory response.

Taken together, the available pharmacologic and physiologic evidence supports a plausible framework in which ticagrelor may increase susceptibility to central respiratory instability through enhanced chemoreflex responsiveness. ENT1 inhibition and reduced adenosine uptake are experimentally demonstrated pharmacologic effects of ticagrelor [13], whereas adenosine-mediated enhancement of chemoreflex activity leading to CSA remains a proposed mechanistic interpretation rather than an established causal pathway in humans. Human studies demonstrating increased ventilatory responses to carbon dioxide and hypoxia provide evidence that ticagrelor exposure can modify respiratory control [3,4]. In a susceptible individual, an exaggerated ventilatory response could promote hyperventilation and hypocapnia, increasing the likelihood that PaCO₂ falls below the apneic threshold during sleep [1]. Repeated ventilatory overshoot followed by central apnea could thereby contribute to periodic breathing and other central respiratory patterns. This sequence, however, remains a mechanistic framework rather than a demonstrated causal pathway in humans; current evidence does not establish that ENT1 inhibition, adenosine signaling, or any single chemoreflex pathway is necessary or sufficient for ticagrelor-associated CSA.

Clinical evidence for ticagrelor-associated CSA

Early evidence linking ticagrelor with central respiratory instability emerged from clinical observations and case reports. Giannoni et al. described Cheyne-Stokes respiration and heightened chemoreflex sensitivity in association with ticagrelor-related dyspnea, raising the possibility that ticagrelor-related dyspnea may accompany an objective disturbance of ventilatory control rather than represent an isolated subjective respiratory symptom [7]. Conte et al. subsequently reported persistent Cheyne-Stokes respiration and heart-rate instability during ticagrelor treatment in a patient with congestive heart failure, with improvement following aminophylline administration [14]. However, heart failure itself is an established cause of periodic breathing and substantially limits causal attribution in that case. Lamberts et al. later documented Cheyne-Stokes respiration during polysomnographic evaluation in a patient receiving ticagrelor [15]. These observations were complemented by a pharmacovigilance analysis by Revol et al. using VigiBase, the World Health Organization global database of individual case safety reports submitted through participating national drug-safety systems [8]. The investigators identified 28 reports of sleep apnea associated with ticagrelor and a significant disproportionality signal relative to the broader database [8]. Because spontaneous pharmacovigilance reports are susceptible to underreporting, selective reporting, incomplete clinical characterization, and uncontrolled confounding, these data cannot establish incidence or causality. Rather, they strengthened an emerging safety signal and provided justification for more systematic sleep and respiratory-physiology investigation.

More persuasive case-level evidence subsequently came from observations in which respiratory abnormalities changed in parallel with ticagrelor exposure. Puel et al. described two patients in whom CSA improved after ticagrelor withdrawal and recurred following reintroduction of the drug [9]. In one patient, the central apnea-hypopnea index decreased from 14 events/hour during ticagrelor exposure to 7 events/hour after substitution with prasugrel and subsequently increased markedly after ticagrelor was reintroduced [9]. Paboeuf et al. reported a complementary pattern in a patient with previously treated obstructive sleep apnea who developed CSA and Cheyne-Stokes respiration after ticagrelor initiation [16]. The change in respiratory phenotype was accompanied by increased hypercapnic chemosensitivity; following ticagrelor withdrawal, the predominantly central pattern resolved and chemosensitivity decreased, while transient central apnea and Cheyne-Stokes respiration recurred after accidental re-exposure [16]. Although these observations involve very small numbers of patients and cannot establish incidence or population-level risk, the reproducibility of respiratory abnormalities during withdrawal and re-exposure strengthens causal inference beyond that provided by temporal association alone.

The evidence base subsequently expanded to comparative physiologic and observational studies. Giannoni et al. evaluated 60 patients with acute coronary syndrome, preserved left ventricular systolic function, and no history of obstructive sleep apnea who were receiving either ticagrelor or prasugrel [3]. Using 24-hour cardiorespiratory monitoring and hypercapnic chemosensitivity testing, the investigators found higher apnea-hypopnea and central apnea indices, together with greater hypercapnic chemosensitivity, among patients receiving ticagrelor [3]. Meurin et al. subsequently prospectively evaluated 121 patients after acute coronary syndrome who had neither heart failure nor a known history of sleep apnea [10]. Predominantly CSA with an apnea-hypopnea index of at least 15 events/hour was identified in 24 of 80 patients receiving ticagrelor (30%) compared with three of 41 patients not receiving ticagrelor (7.3%), and ticagrelor use remained associated with CSA in multivariable analysis [10]. Tzeng et al. later examined 135 patients with coronary heart disease who underwent polysomnography across three sleep centers and found a greater central apnea burden among patients receiving ticagrelor than among those receiving clopidogrel [11]. Ticagrelor remained associated with high central apnea burden after adjustment for age and left ventricular dysfunction [11]. Collectively, these studies extended the initial case-based signal into larger clinical populations and demonstrated that the association remained detectable in analyses that either excluded heart failure or accounted for ventricular dysfunction as an important competing explanation for central apnea.

Not all available evidence has demonstrated an association between ticagrelor and sleep apnea. Sabatine et al. analyzed individual patient data from six large randomized, double-blind phase 3 trials comprising 86,195 participants and examined treatment-emergent adverse events coded as sleep apnea syndrome [12]. Across the pooled trials, ticagrelor was not associated with a significant increase in reported sleep apnea compared with control treatment [12]. This represents important counterevidence because randomized trial data reduce many forms of treatment-selection bias. However, sleep apnea in these trials was identified through adverse-event reporting rather than systematic polysomnography, respiratory polygraphy, or prospective characterization of central respiratory events, and central and obstructive phenotypes were not specifically distinguished. The randomized-trial findings therefore argue against a large readily recognized excess of clinically reported sleep apnea but do not directly resolve whether ticagrelor increases objectively measured central respiratory instability in susceptible patients.

Considered together, the human evidence comprises several complementary but methodologically distinct lines of investigation. Early reports established a temporal clinical signal, while withdrawal and re-exposure observations strengthened causal inference at the individual-patient level [7,9,16]. Pharmacovigilance analysis identified disproportionate reporting but could not determine incidence or adequately control for confounding [8]. Comparative physiologic studies demonstrated altered chemosensitivity and increased central respiratory events during ticagrelor exposure [3,4], while prospective and retrospective sleep studies identified higher central apnea burdens in selected ticagrelor-treated cardiovascular populations undergoing targeted respiratory assessment [10,11]. Conversely, large randomized trial datasets did not demonstrate an excess of reported sleep apnea [12]. These findings are not necessarily directly contradictory because the studies differed substantially in patient selection, respiratory assessment, and outcome definition. The observational and physiologic studies specifically characterized central respiratory events using cardiorespiratory monitoring, polysomnography, or related physiologic testing, whereas the randomized trials relied on treatment-emergent adverse-event reporting of sleep apnea syndrome without systematic respiratory phenotyping or distinction between central and obstructive events [12]. The findings should therefore be interpreted according to the population, ascertainment method, and respiratory endpoint of each study rather than as a uniform evidence base addressing an identical outcome. The principal human clinical, pharmacovigilance, and physiologic studies evaluating ticagrelor-associated central respiratory abnormalities are summarized in Table 1.

**Table 1:** Human clinical, pharmacovigilance, and physiologic evidence regarding ticagrelor-associated central sleep apnea This table summarizes the principal human clinical, pharmacovigilance, and physiologic studies evaluating ticagrelor-associated central respiratory abnormalities. Study designs, populations, respiratory assessments, and outcome definitions differed substantially across reports and should not be interpreted as directly comparable. Case reports and dechallenge-rechallenge observations provide evidence regarding temporality and individual-level causal inference, whereas pharmacovigilance analyses identify reporting signals rather than incidence. Comparative physiologic and observational studies provide broader evidence regarding chemosensitivity and central respiratory-event burden but remain subject to confounding and differences in ascertainment. Accordingly, the findings are presented according to study design and evidentiary context rather than as a quantitatively pooled estimate. Abbreviations: AHI: apnea-hypopnea index; ACS: acute coronary syndrome; CAI: central apnea index; CSA: central sleep apnea; CSR: Cheyne-Stokes respiration; HCVR: hypercapnic ventilatory response; HR: hazard ratio; HVR: hypoxic ventilatory response; LVEF: left ventricular ejection fraction; OSA: obstructive sleep apnea; OR: odds ratio; PAP: positive airway pressure; PSG: polysomnography; WHO: World Health Organization

Study	Population and design	Ticagrelor exposure/comparator	Respiratory assessment	Principal findings	Interpretation and limitations
Giannoni et al. (2016) [7]	Index patient plus three additional patients evaluated after acute coronary syndrome	Ticagrelor; withdrawal in the index patient	24-hour cardiorespiratory monitoring and hypercapnic chemosensitivity testing	Cheyne-Stokes respiration and increased respiratory-event burden were observed during ticagrelor exposure. In the index patient, withdrawal was followed by progressive disappearance of Cheyne-Stokes respiration and reduction in hypercapnic chemosensitivity. Similar respiratory patterns were observed in three additional patients.	Foundational clinical and physiologic signal. Small uncontrolled report; post-acute coronary syndrome status remains a potential confounder, although reported cardiopulmonary function was preserved.
Conte et al. (2017) [14]	Single patient with congestive heart failure and non-ST-segment elevation myocardial infarction	Ticagrelor; aminophylline administered during adverse events	Clinical respiratory and cardiac monitoring	Persistent Cheyne-Stokes respiration associated with heart-rate instability was reported during ticagrelor treatment, with improvement following aminophylline administration.	Supports a possible adenosine-related contribution, but heart failure is itself a major cause of Cheyne-Stokes respiration and substantially limits causal attribution.
Lamberts et al. (2018) [15]	Single patient receiving ticagrelor after myocardial infarction	Ticagrelor followed by substitution with clopidogrel	Polysomnography	Cheyne-Stokes respiration was documented during ticagrelor exposure and disappeared after drug substitution.	Objective polysomnographic evidence with dechallenge, but limited to an individual case.
Revol et al. (2018) [8]	WHO VigiBase pharmacovigilance case-noncase analysis	Ticagrelor compared with the broader pharmacovigilance database and other antiplatelet agents	Spontaneous adverse-event reports	Twenty-eight sleep-apnea reports were identified with ticagrelor, with a reporting odds ratio of 4.16 (95% CI 2.87-6.03). Comparable disproportionality was not identified for clopidogrel, prasugrel, or aspirin.	Establishes a pharmacovigilance signal rather than incidence or causality; susceptible to reporting bias, incomplete phenotyping, and confounding.
Puel et al. (2019) [9]	Two patients with central respiratory events during ticagrelor treatment	Ticagrelor withdrawal/substitution with prasugrel followed by ticagrelor reintroduction	Polysomnography and positive airway pressure device monitoring	Central respiratory events decreased after ticagrelor withdrawal and recurred after reintroduction. In one patient, the central apnea-hypopnea index decreased from 14/h to 7/h after substitution with prasugrel before worsening again after ticagrelor re-exposure.	Strong dechallenge-rechallenge evidence at the individual-patient level, but based on only two cases.
Paboeuf et al. (2019) [16]	Single patient with previously treated obstructive sleep apnea and preserved left ventricular ejection fraction	Ticagrelor initiation, withdrawal, and accidental re-exposure	Positive airway pressure telemonitoring, respiratory polygraphy, and hypercapnic ventilatory response	Obstructive sleep apnea shifted to a predominantly central/Cheyne-Stokes pattern during ticagrelor exposure with increased hypercapnic chemosensitivity. Withdrawal was followed by return to predominantly obstructive events and normalization of the hypercapnic ventilatory response; accidental re-exposure produced transient recurrence of central sleep apnea/Cheyne-Stokes respiration.	Particularly informative because respiratory phenotype and chemosensitivity varied with drug exposure, but remains single-patient evidence.
Sabatine et al. (2020) [12]	Pooled individual-patient analysis of six randomized, double-blind phase 3 trials; 86,195 participants	Ticagrelor versus randomized control therapy	Treatment-emergent adverse-event reporting	Among 158 reported sleep-apnea adverse events, ticagrelor was not associated with increased risk compared with control treatment (HR 0.82, 95% CI 0.60-1.12).	Important discordant evidence from very large randomized populations; sleep apnea was not systematically assessed by polysomnography and central versus obstructive phenotypes were not distinguished.
Giannoni et al. (2021) [3]	60 patients with acute coronary syndrome, preserved left ventricular ejection fraction, and no history of obstructive sleep apnea; 30 ticagrelor and 30 prasugrel	Ticagrelor 90 mg twice daily versus prasugrel 10 mg daily	24-hour cardiorespiratory monitoring and hypercapnic chemosensitivity testing	Ticagrelor-treated patients demonstrated higher daytime, nighttime, and 24-hour apnea-hypopnea and central apnea indices together with greater hypercapnic chemosensitivity.	Comparative human evidence linking ticagrelor with central respiratory events and altered chemoreflex responsiveness; not formal polysomnography and treatment allocation was observational.
Meurin et al. (2021) [10]	Prospective cohort of 121 patients within one year after acute coronary syndrome without heart failure or known sleep apnea	80 receiving ticagrelor; 41 not receiving ticagrelor	Overnight sleep study	Predominantly central sleep apnea with apnea-hypopnea index ≥15/h occurred in 24/80 (30%) ticagrelor-treated patients versus 3/41 (7.3%) patients not receiving ticagrelor; ticagrelor remained associated with central sleep apnea in multivariable analysis.	Important prospective evidence with heart failure excluded, although exposure was nonrandomized and the cohort was limited to post-acute coronary syndrome patients.
Tubek et al. (2023) [4]	Prospective proof-of-concept study; 11 ticagrelor-treated and 11 matched clopidogrel-treated patients	Measurements before and approximately four weeks after treatment initiation	Hypoxic and hypercapnic ventilatory-response testing	Ticagrelor significantly increased both hypoxic and hypercapnic ventilatory responses, whereas clopidogrel did not significantly alter either measure.	Direct human evidence for increased peripheral and central chemoreflex responsiveness, but sleep and central sleep apnea outcomes were not measured.
Tzeng et al. (2024) [11]	Multicenter retrospective cohort of 135 patients with coronary heart disease undergoing clinically indicated polysomnography; 26 ticagrelor and 109 clopidogrel	Ticagrelor versus clopidogrel	Full polysomnography at three sleep centers	High central sleep apnea burden occurred in 27% versus 8.3% with ticagrelor versus clopidogrel. Strict central sleep apnea was observed in 4 of 26 ticagrelor-treated patients and 2 of 109 clopidogrel-treated patients. Ticagrelor remained associated with high central sleep apnea burden after adjustment for age and left ventricular dysfunction.	Multicenter polysomnographic evidence, but retrospective design, clinically selected population, small ticagrelor subgroup, and residual confounding limit causal inference.

As summarized individually in Table 1, the methodological strength of the available evidence varies considerably by study design. Case reports and dechallenge-rechallenge observations provide useful individual-level temporal evidence but are limited by small sample size, lack of control groups, and limited generalizability. Pharmacovigilance analyses are useful for signal detection but are vulnerable to reporting bias and cannot establish incidence or causality. Comparative observational studies provide stronger population-level evidence but remain susceptible to treatment-selection bias, cardiovascular confounding, and differences in respiratory-event ascertainment. Physiologic studies contribute mechanistic support through assessment of ventilatory chemosensitivity and related respiratory-control responses, although these measures are not equivalent to clinically defined CSA. Randomized trial data provide the strongest protection against treatment-selection bias, but their relevance to this specific question is limited when sleep apnea is identified through adverse-event reporting rather than standardized respiratory phenotyping. Accordingly, the overall literature should be interpreted by weighing each study according to both design strength and the specificity of the respiratory outcome measured.

Clinical implications

Recognition of ticagrelor-associated CSA is clinically relevant because respiratory symptoms during ticagrelor therapy may initially be attributed to the well-recognized adverse effect of dyspnea. Dyspnea occurs more frequently with ticagrelor than with clopidogrel, but it is a nonspecific symptom and does not, by itself, establish the presence of central sleep-disordered breathing [6]. The American Academy of Sleep Medicine recommends that CSA be evaluated within the individual clinical context, including contributing conditions, comorbidities, symptoms, and polysomnographic findings, with management directed toward the underlying cause rather than respiratory-event suppression alone [17]. In patients receiving ticagrelor, suspicion for a medication-associated central component may therefore be warranted when new respiratory symptoms are accompanied by witnessed periodic breathing, nocturnal respiratory pauses, unexplained sleep fragmentation or daytime sleepiness, or the emergence of central events on positive airway pressure device data. Ticagrelor-associated central respiratory abnormalities have also been observed in populations without overt heart failure or markedly reduced left ventricular systolic function [3,10,11]. Accordingly, ticagrelor should be considered during medication review when otherwise unexplained central respiratory events develop during treatment, while alternative causes of CSA remain under consideration.

Once a medication-associated contribution is suspected, evaluation should still address the broader differential diagnosis rather than attributing central events to ticagrelor on the basis of temporal association alone. Potential competing contributors include heart failure, neurologic disease, opioid or other medication exposure, high-altitude exposure, and other conditions capable of altering respiratory drive or ventilatory stability [1,2]. Objective sleep testing is therefore important when symptoms, witnessed breathing abnormalities, or positive airway pressure device data suggest a substantial central component. The ticagrelor literature itself illustrates the value of physiologic characterization, as published investigations have used methods ranging from cardiorespiratory monitoring and respiratory polygraphy to full polysomnography [3,9-11,15,16]. Polysomnography can help distinguish predominantly central from obstructive respiratory events, quantify event burden, and identify coexisting sleep-disordered breathing. Measurement of ventilatory chemosensitivity has provided additional mechanistic insight in research studies [3,4,7,16] but is not routinely required for clinical evaluation of suspected medication-associated CSA.

When the temporal relationship and objective sleep findings support a possible ticagrelor-associated effect after consideration of competing causes, management should balance the significance of the respiratory abnormality against the cardiovascular indication for antiplatelet therapy. Several reports have documented improvement in central respiratory events after ticagrelor withdrawal or substitution with another P2Y12 inhibitor, with recurrence following re-exposure in some patients [9,15,16]. These observations support medication modification as a potentially informative and therapeutic intervention when clinically appropriate, but they do not establish a standardized treatment strategy or justify routine discontinuation of ticagrelor whenever CSA is identified. Because ticagrelor is prescribed to reduce ischemic cardiovascular risk, any change in antiplatelet therapy should be coordinated with the clinician managing the underlying cardiovascular disease [5]. When ticagrelor cannot be discontinued, or when central respiratory events persist despite withdrawal, treatment should be guided by the residual CSA phenotype, associated comorbidities, symptoms, and objective sleep-study findings rather than assuming that medication exposure is the sole determinant of respiratory instability [17].

In practical terms, evaluation can follow a stepwise approach: suspect a ticagrelor-associated contribution when new central respiratory symptoms or events emerge during treatment; obtain objective sleep testing when a clinically meaningful central component is suspected; evaluate competing causes of CSA; and consider modification of ticagrelor only when the temporal relationship and objective findings are persuasive. Any change in antiplatelet therapy should be coordinated with the clinician managing the underlying cardiovascular disease, and routine discontinuation of ticagrelor is not supported by the current evidence.

More broadly, ticagrelor-associated central respiratory instability may illustrate a form of medication-associated sleep-disordered breathing characterized by excessive ventilatory responsiveness rather than conventional respiratory suppression. This distinction is clinically relevant because medication-induced CSA is commonly considered in the context of agents that depress respiratory rhythm generation, whereas ticagrelor has instead been associated with increased chemosensitivity and ventilatory instability [2-4]. The observation that ticagrelor has also been associated with periodic breathing during exercise suggests that the underlying disturbance in respiratory control may not be confined to sleep [18]. However, the pharmacologic pathway responsible for this phenotype remains uncertain. Although inhibition of adenosine uptake through ENT1 provides a plausible upstream mechanism [13], plasma adenosine concentrations did not differ between patients with and without ticagrelor-related dyspnea in a human pharmacologic study [19]. Ticagrelor-associated CSA should therefore be viewed as an emerging clinical association supported by converging clinical and physiologic observations, while the specific molecular mechanisms and determinants of individual susceptibility remain incompletely defined.

Limitations and future directions

Several limitations constrain interpretation of the current evidence regarding ticagrelor-associated CSA. Most importantly, the direct human evidence base remains limited and methodologically heterogeneous. The initial literature consists largely of individual case reports and small case series, including observations in which central respiratory abnormalities improved after ticagrelor withdrawal and recurred following re-exposure [7,9,15,16]. These reports provide valuable evidence regarding temporality and individual-level causal inference but cannot establish incidence, quantify population-level risk, or identify which patients are most susceptible. Larger studies have subsequently provided comparative and observational evidence, but they differ substantially in patient selection, cardiovascular status, comparator treatment, respiratory methodology, and outcome definitions [3,10,11]. Giannoni et al. studied patients with preserved left ventricular systolic function [3], Meurin et al. excluded patients with heart failure [10], and Tzeng et al. adjusted for age and left ventricular dysfunction [11]. These approaches reduce, but do not eliminate, cardiovascular confounding. Acute coronary syndromes, heart failure, and ventricular dysfunction may independently alter respiratory control and contribute to CSA or Cheyne-Stokes respiration. Residual confounding therefore remains an important limitation when interpreting the association between ticagrelor exposure and central respiratory instability.

The available studies also differ substantially in how respiratory abnormalities were identified and classified. Published investigations have used full polysomnography, respiratory polygraphy, prolonged cardiorespiratory monitoring, positive airway pressure device data, spontaneous adverse-event reports, and laboratory measurements of ventilatory chemosensitivity [3,4,8-12,15,16]. These approaches are not interchangeable. An adverse-event diagnosis of sleep apnea does not provide the same physiologic information as polysomnographic determination of central apnea burden, while measurements of hypercapnic or hypoxic ventilatory responsiveness characterize respiratory-control physiology rather than sleep apnea itself. This distinction is particularly important when interpreting the absence of increased sleep-apnea reporting in pooled randomized ticagrelor trials [12] alongside positive findings from studies that systematically characterized central respiratory events [3,10,11]. Definitions have also varied across studies, including predominantly CSA, high central apnea burden, Cheyne-Stokes respiration, and central apnea indices derived from different monitoring methods and intervals. Thresholds used to define clinically significant sleep apnea or high central apnea burden also differed across studies, further limiting direct comparison of prevalence estimates and effect sizes. Night-to-night variability may further complicate characterization of central respiratory instability. The available literature therefore does not support treating all reported respiratory outcomes as equivalent manifestations of a single uniformly defined phenotype.

Mechanistic interpretation remains similarly constrained. Human physiologic studies provide evidence that ticagrelor exposure can increase hypercapnic and hypoxic ventilatory responsiveness [3,4], supporting enhanced chemosensitivity as one plausible contributor to respiratory instability. However, increased chemosensitivity is neither synonymous with CSA nor sufficient to establish the sequence by which central events develop in an individual patient. Existing studies have not established whether ticagrelor consistently reduces the carbon dioxide reserve, increases overall loop gain, alters the apneic threshold, or modifies other components of ventilatory control during sleep. Likewise, ticagrelor inhibits ENT1 and can alter extracellular adenosine handling [13], but these pharmacologic observations provide biological plausibility rather than proof that adenosine signaling mediates central apnea in humans. Human data showing no simple relationship between circulating adenosine concentrations and ticagrelor-related dyspnea further argue against reducing the respiratory phenotype to a single measurable adenosine pathway [19]. The relative contributions of ENT1 inhibition, P2Y12-related signaling, central and peripheral chemoreflex pathways, cardiovascular physiology, and other mechanisms therefore remain unresolved.

The determinants of individual susceptibility are also poorly defined. The available literature does not establish the proportion of ticagrelor-treated patients who develop clinically recognized CSA or why central respiratory instability becomes prominent in some individuals. Potential modifiers may include baseline ventilatory chemosensitivity, loop gain, proximity of waking or sleeping PaCO₂ to the apneic threshold, underlying obstructive sleep apnea, age, ventricular function, acute versus stable cardiovascular disease, concomitant medications, and other physiologic characteristics. These factors should be regarded as potential susceptibility modifiers rather than established predictors because no prospective study has validated a clinical risk profile for ticagrelor-associated CSA. Existing studies have generally not been designed or powered to determine the independent contribution of these factors or their interactions. The observation of ticagrelor-associated periodic breathing during exercise further raises the possibility that susceptibility reflects a broader ventilatory-control phenotype rather than an abnormality restricted to sleep [18]. Whether patients exhibiting respiratory instability during wakefulness or exercise are also those most likely to develop central events during sleep remains unknown.

The clinical significance of ticagrelor-associated central respiratory abnormalities is likewise incompletely established. Published studies have primarily characterized respiratory-event frequency, breathing pattern, or chemosensitivity rather than prospectively linking these abnormalities to patient-important outcomes. It therefore remains uncertain whether ticagrelor-associated central apnea independently contributes to impaired sleep quality, excessive daytime sleepiness, cognitive or functional impairment, cardiovascular morbidity, medication discontinuation, or other adverse clinical outcomes. Accordingly, the presence of ticagrelor-associated central respiratory events should not be assumed to imply clinically meaningful harm in the absence of demonstrated effects on symptoms, sleep quality, daytime function, cardiovascular outcomes, or treatment tolerance. Conversely, the presence of objectively measured central events should not automatically be interpreted as evidence that ticagrelor withdrawal will provide a net clinical benefit, particularly because the drug may be prescribed for an important cardiovascular indication. Dechallenge and rechallenge reports demonstrate that the respiratory phenotype may be reversible in selected patients [9,15,16], but they do not define an event-burden threshold at which modification of antiplatelet therapy becomes appropriate or establish whether treatment of the respiratory abnormality improves clinically meaningful outcomes. Future studies should therefore distinguish demonstration of a physiologic drug effect from demonstration of clinical harm.

Future investigation should prioritize adequately powered prospective studies using standardized respiratory phenotyping and clearly defined ticagrelor exposure. Where feasible, participants should undergo baseline polysomnography before ticagrelor initiation and repeat polysomnography after treatment begins so that pre-existing central respiratory abnormalities can be distinguished from treatment-emergent changes. Appropriate comparator groups receiving other P2Y12 inhibitors, such as clopidogrel or prasugrel, would help separate ticagrelor-specific effects from respiratory changes associated with the underlying cardiovascular disease. Standardized polysomnographic scoring should characterize obstructive and central apneas separately and, where feasible, distinguish central from obstructive hypopneas. Serial or multinight measurements could help determine the reproducibility and persistence of the phenotype. Standardized assessment of hypercapnic and hypoxic ventilatory chemosensitivity should be incorporated when feasible to determine whether changes in respiratory-control physiology precede or accompany the development of central respiratory events. Concurrent assessment of PaCO₂ or appropriate surrogate measures, ventricular function, medication exposure, symptoms, subjective sleep quality, daytime functioning, and cardiovascular outcomes would permit mechanistic observations to be related to clinically meaningful consequences. Patient-centered outcomes, including sleep quality, daytime sleepiness, functional impairment, treatment tolerance, medication discontinuation, and cardiovascular events, should be assessed prospectively rather than relying solely on respiratory-event indices. Expert recommendations for CSA research similarly emphasize improved respiratory-event phenotyping, attention to temporal variability, mechanistic characterization, and outcomes extending beyond the apnea-hypopnea index alone [20]. Such studies could clarify incidence, exposure-response relationships, reversibility, susceptibility factors, and whether ticagrelor-associated CSA represents a clinically consequential adverse drug effect in a definable subgroup.

Finally, this article is a narrative review rather than a systematic review or meta-analysis. Although the literature-search and evidence-selection approach was structured to improve transparency, no formal risk-of-bias instrument was applied, and qualitative selection and synthesis remain susceptible to selection bias. This consideration is particularly important for the mechanistic literature because pharmacologic and physiologic studies were selected for their relevance to proposed pathways; prominence of ENT1 inhibition, adenosine signaling, or chemoreflex activation within the contextual literature should therefore not be interpreted as evidence that any one mechanism has been demonstrated to mediate ticagrelor-associated CSA in humans. The direct clinical studies also differ sufficiently in design, populations, respiratory ascertainment, and outcome definitions that quantitative pooling would risk combining noncomparable endpoints. In addition, reliance on English-language literature identified principally through PubMed, supplemented by Google Scholar and citation tracking, may introduce language, database-selection, and discretionary-selection bias and may omit relevant evidence indexed elsewhere. These methodological limitations, together with the limited and heterogeneous direct evidence base, require that conclusions remain proportional to the available data.

## Conclusions

The available human evidence supports an association between ticagrelor exposure and central respiratory instability, including CSA, Cheyne-Stokes respiration, and increased ventilatory chemosensitivity. Evidence is derived from case reports with dechallenge-rechallenge observations, comparative physiologic studies, prospective and retrospective clinical cohorts, pharmacovigilance data, and polysomnographic investigations. However, the evidence base remains heterogeneous, and large randomized trial datasets based on adverse-event reporting have not demonstrated an excess of reported sleep apnea. Ticagrelor-associated CSA should therefore be regarded as an emerging medication-associated respiratory phenotype rather than an established population-wide adverse effect with a defined incidence.

The available physiologic evidence points toward increased ventilatory responsiveness and susceptibility to respiratory instability rather than conventional respiratory suppression. ENT1 inhibition and altered adenosine signaling provide biological plausibility, but no specific molecular pathway has been demonstrated to mediate CSA in humans. Clinically, ticagrelor should be considered during medication review when otherwise unexplained central respiratory events develop, while competing causes of CSA are carefully evaluated and the cardiovascular indication for antiplatelet therapy is considered in any treatment decision. Larger prospective studies using standardized polysomnographic phenotyping, appropriate comparator groups, serial assessment, and clinically meaningful outcomes are needed to define incidence, susceptibility factors, reversibility, mechanistic pathways, and the clinical significance of ticagrelor-associated central respiratory abnormalities.
